# Editorial: Biological drugs and biosimilars in autoimmune diseases

**DOI:** 10.3389/fphar.2023.1168972

**Published:** 2023-03-02

**Authors:** Anna Wajda, Carlo Perricone, Anniruddh Kashyap, Mahmud Yerima Iliyasu

**Affiliations:** ^1^ Department of Molecular Biology, National Institute of Geriatrics, Rheumatology and Rehabilitation, Warsaw, Poland; ^2^ Rheumatology Unit, Department of Medicine and Surgery, University of Perugia, Perugia, Italy; ^3^ Department of Laboratory Medicine, Örebro University Hospital, Örebro, Sweden; ^4^ Department of Biological Sciences, Faculty of Science, Abubakar Tafawa Balewa University, Bauchi, Nigeria

**Keywords:** biologics, biosimilar, autoimmune disease, rheumatology and immunology, allergology and immunology

The usage of biological drugs in autoimmune diseases treatment has significantly changed the clinical disease course especially for patients with particularly complicated or poor response to the treatment, demonstrating the ability to inhibit disease progression. Therefore, nowadays, biologics represent an increasing proportion of total pharmaceutical expenses despite differences in standard of routine care and high prices (Reardon, 2020). The articles presented in the Research Topic highlight the broad area of this subject. In this Research Topic, three reviews, a case report, a research report and an original research articles were published.

It has been over 20 years since the first clinical trials of anti-TNF alpha therapy (Elliott et al., 2008). Currently available biological drugs for clinical use in rheumatology are adalimumab, golimumab, infliximab, certolizumab, and etanercept. Adalimumab and golimumab are fully human monoclonal antibodies; infliximab is a chimeric monoclonal antibody with a murine variable region; certolizumab is a humanized Fab fragment conjugated with polyethylene glycol, while etanercept is a fusion protein of two TNF2 receptor extracellular domains and the Fc fragment of human immunoglobulin. Since then, anti-TNF therapy has found applications in various autoimmune diseases. Some of these are very rare, such as Behçet’s disease (BD). A systematic review of controlled trials of anti-TNF therapy compared with conventional therapy was presented by Zhou et al.

The authors investigated the efficacy and safety of anti-TNF therapy and summarized it in relation to the available therapeutic options. According to the authors, anti-TNF therapy reduces the relapses of uveitis in patients with BD in comparison with the traditional immunosuppressant (csDMARDs) therapy. This finding has significant implications for the protection against significant and permanent vision impairment or blindness. However, it has to be taken into consideration, that BD is a heterogeneous disease and presents different subtypes, such as Behçet’s uveitis, intestinal BD, neurological-BD, and vascular-BD. Moreover, Zhou et al. found that both IFNα-2α and infliximab therapy (anti-TNF agent) were effective in inducing uveitis remission in patients with BD and that adalimumab and infliximab provided similar types of trends and qualitative conclusions in two studies on Behçet’s uveitis.

Two decades of presence on the medical market also allowed an evaluation of the side effects of anti-TNF therapy. Li et al. presented a meta-analysis in which it was revealed that treatment with anti-TNF agents resulted in a significant increase in the risk of serious infections and a bit lower, but still significant, increased risk of cancer. On the other hand, the risk of developing tuberculosis was not significantly different between anti-TNF agents *versus* those other non-anti-TNF agents. The analysis by Li et al. focused on patients with rheumatoid arthritis (RA), psoriatic arthritis (PsA), and ankylosing spondylitis (AS).

Wen et al. focused their attention on the interaction between anti-TNF agents and other drugs in an illustrative case report. The authors noted the interaction between etanercept and cyclosporine (CsA) in a 42-years-old male patient with ankylosing spondylitis and IgA nephropathy. Physicians concluded that the use of etanercept led to at least a 2.5-fold increase in total CsA clearance presumably with IL-2 involvement. Despite the fact that biologic drugs are not metabolised by CYP-isoenzymes neither excreted by active transporters, and there are no reported pharmacokinetic interactions with other drugs involving liver enzymes and transport proteins, it is likely that some biologics may affect CYP-enzyme expression (Pflugbeil et al., 2020). Particularly, drug concentration of CsA should be monitored during initiation and discontinuation of adalimumab therapy, as adalimumab initiation could increase CYP synthesis and lead to decreased CsA concentrations. The authors of this case study warn against similar interactions and recommend monitoring cytokine levels if a drug-cytokine interaction is suspected.

Olewicz-Gawlik and Kowala-Piaskowska presented a review on IgE-mediated autoimmunity and anti-IgE treatment. IgE is recognised as a mediator of an immediate, allergen-specific immune response known as type 1 hypersensitivity. However, the early reports in the late 70s of the XX century about the presence of anti-nuclear IgE antibodies in patients with autoimmune diseases changed this perception. The authors pointed out that the mechanisms leading to a preference in some B-cells for switching to autoantibodies of IgE isotype are still unclear. Nevertheless, they summarized newly recognised mechanisms implicating IgE in the pathogenesis of autoimmune diseases. To date, the only anti-IgE therapy approved for clinical use is omalizumab, a recombinant humanized monoclonal antibody. Effective results of omalizumab have been noted in systemic lupus erythematosus (SLE), bullous pemphigoid, chronic spontaneous urticaria, pemphigus vulgaris and atopic dermatitis. In the patients with chronic spontaneous urticaria, another anti-IgE agent - ligelizumab, with ongoing clinical trials, revealed promising results of the treatment. On the other hand, there are reports showing the possible development of other autoimmune diseases in the course of omalizumab treatment. Probably, further studies on omalizumab and ligelizumab in autoimmune diseases will clarify the mechanism of anti-IgE therapy.

Another therapeutic strategy in inflammatory diseases is based on JAK/STAT inhibitors, such as ruxolitinib. Huarte et al. proved that ruxolitinib significantly reduces the harmful consequences of immune overactivation in multiple hyperinflammatory models. In comparison to monoclonal antibody therapy targeting a single cytokine, ruxolitinib can effectively decrease the functional impact of multiple cytokines implicated in hyperactivation states, without widespread immunosuppression. The authors support the hypothesis that JAK/STAT-pathway activation plays a critical role in hyperinflammatory syndromes and that its pharmacological inhibition may represent a viable therapeutic strategy.

Last but not least, Eiza et al. presented an original research on semaphorin 3A as a potential regulatory molecule for establishing immune homeostasis in immune-mediated diseases, such as RA or SLE. Semaphorin 3A, belongs to a large family of transmembrane and secreted phylogenetically conserved proteins, which inhibits T-cell proliferation, migration, and pro-inflammatory cytokine secretion and reconstructs the B-cell regulatory function by expression of CD72. The authors generated a stable and modified molecule, able to activate CD72, sema3A (called Truncated-sema3A, T-sema3A) to avoid its unwanted binding to Neuropilin-1 (NRP-1) receptor on endothelial and neuronal cells. T-sema3A is characterized by the lack of the NRP-1 binding domain (the C-terminal site) and has an artificial dimerization site at position 257 (serine residue was exchanged with a cysteine residue). Modified protein induced the expression of IL-10 and TGF-β and downregulated the secretion of pro-inflammatory cytokines such as IL-6, IFN-γ, and IL-17A from human T- and B-lymphocytes.

To conclude, the availability of biosimilars that reduced the treatment costs has once again changed the treatment scenario in autoimmune diseases. Certainly, more studies are required to understand the mechanisms of existing differences in efficacy and side events with originators/biologics in naïve and switching patients.
